# Correction to: Identifying miRNA synergism using multiple-intervention causal inference

**DOI:** 10.1186/s12859-020-3369-1

**Published:** 2020-01-29

**Authors:** Junpeng Zhang, Vu Viet Hoang Pham, Lin Liu, Taosheng Xu, Buu Truong, Jiuyong Li, Nini Rao, Thuc Duy Le

**Affiliations:** 1 Center for Informational Biology, School of Life Science and Technology, University of Electronic Science and Technology of China, Chengdu, 610054 Sichuan China; 2 School of Engineering, Dali University, Dali, 671003 Yunnan China; 3 School of Information Technology and Mathematical Sciences, University of South Australia, Mawson Lakes, SA 5095 Australia; 4 Institute of Intelligent Machines, Hefei Institutes of Physical Science, Chinese Academy of Sciences, Hefei, China; 5 Pham Ngoc Thach University of Medicine, Ho Chi Minh, Vietnam

**Correction to: BMC Bioinformatics (2019) 20(Suppl 23): 613**

**https://doi.org/10.1186/s12859-019-3215-5**

After publication of this supplement article [1], it was brought to our attention that the Fig. 3 was incorrect. The correct Fig. 3 is as below:

**Fig. 3:**
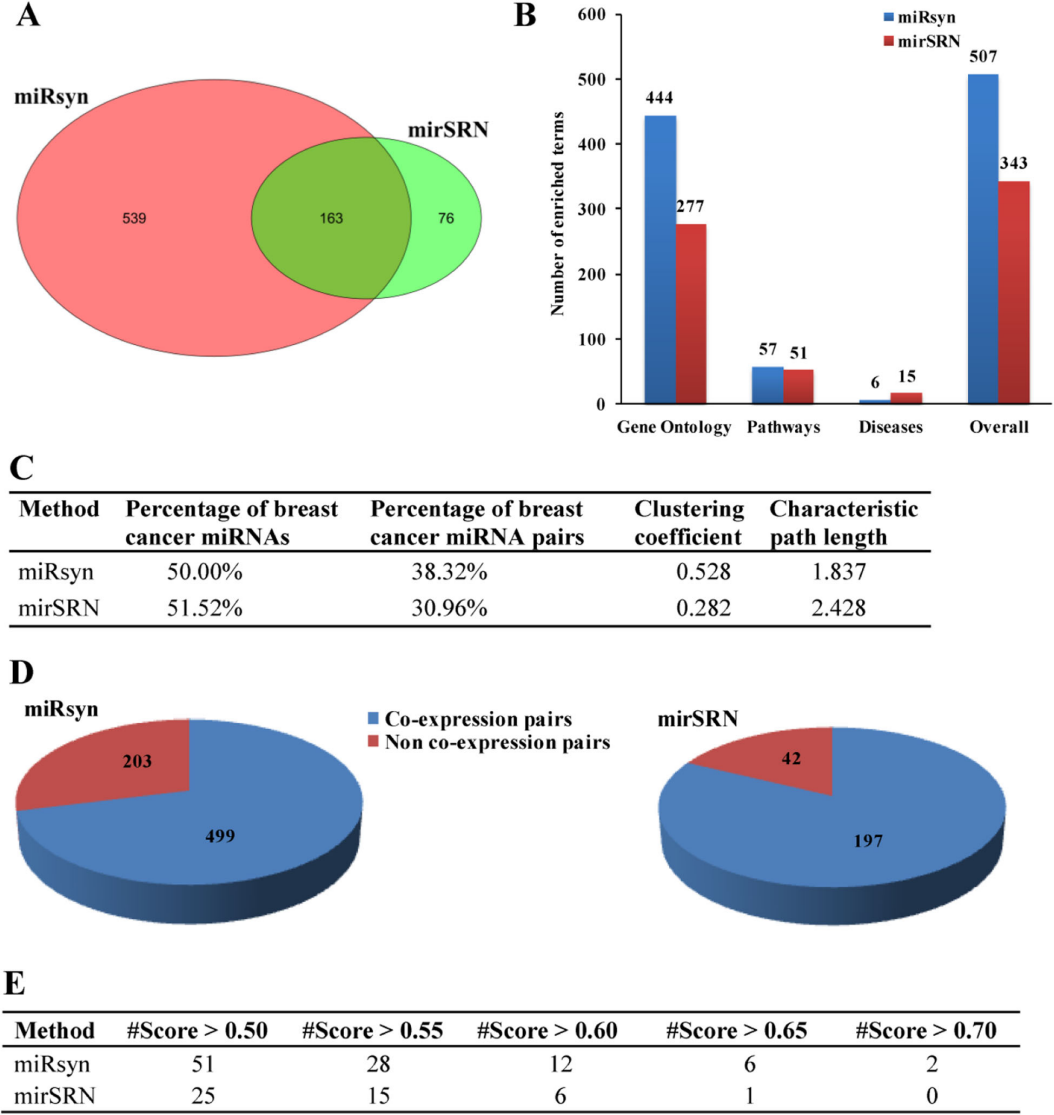
Comparison results between miRsyn and mirSRN. **a** The number of miRNA synergistic pairs. **b** The number of significantly enriched terms. **c** The percentage of breast cancer miRNAs and miRNA synergistic pairs, clustering coefficient and characteristic path length. **d** The number of co-expression and non co-expression miRNA synergistic pairs. **e** The overlap with putative miRNA synergistic pairs under different score cutoffs
